# Plasma Predictive Features in Treating EGFR-Mutated Non-Small Cell Lung Cancer

**DOI:** 10.3390/cancers12113179

**Published:** 2020-10-29

**Authors:** Christi M. J. Steendam, G. D. Marijn Veerman, Melinda A. Pruis, Peggy Atmodimedjo, Marthe S. Paats, Cor van der Leest, Jan H. von der Thüsen, David C. Y. Yick, Esther Oomen-de Hoop, Stijn L. W. Koolen, Winand N. M. Dinjens, Ron H. N. van Schaik, Ron H. J. Mathijssen, Joachim G. J. V. Aerts, Hendrikus Jan Dubbink, Anne-Marie C. Dingemans

**Affiliations:** 1 Department of Pulmonology, Erasmus MC Cancer Institute, University Medical Center, 3015 GD Rotterdam, The Netherlands; c.steendam@erasmusmc.nl (C.M.J.S.); m.pruis@erasmusmc.nl (M.A.P.); m.paats@erasmusmc.nl (M.S.P.); j.aerts@erasmusmc.nl (J.G.J.V.A.); 2 Department of Pulmonology, Amphia Hospital, 4818 CK Breda, The Netherlands; cvanderleest@amphia.nl; 3 Department of Medical Oncology, Erasmus MC Cancer Institute, University Medical Center, 3015 GD Rotterdam, The Netherlands; g.veerman@erasmusmc.nl (G.D.M.V.); e.oomen-dehoop@erasmusmc.nl (E.O.-d.H.); s.koolen@erasmusmc.nl (S.L.W.K.); a.mathijssen@erasmusmc.nl (R.H.J.M.); 4 Department of Pathology, Erasmus MC Cancer Institute, University Medical Center, 3015 GD Rotterdam, The Netherlands; p.atmodimedjo@erasmusmc.nl (P.A.); j.vonderthusen@erasmusmc.nl (J.H.v.d.T.); w.dinjens@erasmusmc.nl (W.N.M.D.); 5 Department of Pathology, Amphia Hospital, 4818 CK Breda, The Netherlands; dyick@amphia.nl; 6 Department of Clinical Chemistry, Erasmus MC Cancer Institute, University Medical Center, 3015 GD Rotterdam, The Netherlands; r.vanschaik@erasmusmc.nl; 7 Department of Pulmonology, Maastricht UMC+, 6229 HX Maastricht, The Netherlands

**Keywords:** NSCLC, EGFR, TKI, cfDNA, plasma conversion, TP53 mutation, T790M mutation, pharmacokinetics

## Abstract

**Simple Summary:**

Patients with non-small cell lung cancer with an activating *EGFR* mutation in the tumor, are treated with targeted therapy against this mutation. In the end, all patients develop resistance against this therapy, but some patients have a very short or no benefit. In this study, the authors used blood samples from 41 patients to investigate predictive factors for lack of or short efficacy of targeted therapy. They found that lack of disappearance of the treated mutation in blood after 6 or 12 weeks, the presence of co-occurring *TP53* mutations, and decrease of erlotinib therapy concentrations in time are correlated to a shorter time of benefit. Confirmation of these findings in a larger cohort is desirable, this may lead to implementation of blood sampling for DNA analysis and therapy concentration measurement in daily practice in the future, to identify patients in need of closer follow-up or more extensive treatment.

**Abstract:**

Although epidermal growth factor receptor (EGFR)-tyrosine kinase inhibitors (TKIs) are the preferred treatment for patients with *EGFR*-mutated non-small cell lung cancer (NSCLC), not all patients benefit. We therefore explored the impact of the presence of mutations found in cell-free DNA (cfDNA) and TKI plasma concentrations during treatment on progression-free survival (PFS). In the prospective START-TKI study blood samples from 41 patients with *EGFR*-mutated NSCLC treated with EGFR-TKIs were available. Next generation sequencing (NGS) on cfDNA was performed, and plasma TKI concentrations were measured. Patients without complete plasma conversion of *EGFR* mutation at week 6 had a significantly shorter PFS (5.5 vs. 17.0 months, *p* = 0.002) and OS (14.0 vs. 25.5 months, *p* = 0.003) compared to patients with plasma conversion. In thirteen (second line) osimertinib-treated patients with a (plasma or tissue) concomitant *TP53* mutation at baseline, PFS was significantly shorter compared to six wild-type cases; 8.8 vs. 18.8 months, *p* = 0.017. Erlotinib C_mean_ decrease of ≥10% in the second tertile of treatment was also associated with a significantly shorter PFS; 8.9 vs. 23.6 months, *p* = 0.037. We obtained evidence that absence of plasma loss of the primary *EGFR* mutation, isolated plasma p.T790M loss after six weeks, baseline concomitant *TP53* mutations, and erlotinib C_mean_ decrease during treatment are probably related to worse outcome.

## 1. Introduction

Non-small cell lung cancer (NSCLC) has the highest mortality among solid tumors and once metastasized, patients have a limited prognosis and depend on palliative treatment [1]. Lung adenocarcinoma comprises some specific subgroups defined by oncogenic driver mutations, including the epidermal growth factor receptor (*EGFR*)-mutation. The outcome of patients with *EGFR*-mutated NSCLC has significantly improved with the introduction of EGFR-tyrosine kinase inhibitors (TKIs), with a median overall survival (OS) of more than 3 years [2]. However, up to 10% of patients have an initial lack of response (primary resistance) and 10 to 30% of patients have early progressive disease within 6 months [3,4]. Therefore, it is important to identify these patients early, in order to implement close monitoring and immediate switch to a next line of treatment.

Nevertheless, predictive biomarkers for *EGFR*-mutated NSCLC are scarce. Some studies have observed that concomitant mutations are associated with worse clinical outcome [5,6]. Especially, the presence of concomitant *TP53* mutations was associated with shorter progression-free survival (PFS) and OS [7,8,9,10].

Next to tumor mutational characteristics, treatment outcome might be dependent on the actual TKI exposure. For multiple TKIs relationships have been found between pharmacokinetic parameters (e.g., minimal drug concentration or total exposure) and OS or PFS [11]. Variability in TKI exposure is high and can be influenced by drug–drug interactions, genetic variations in drug metabolizing (CYP) enzymes, lifestyle (e.g., smoking), and concomitant intake of food or herbs [12,13]. This is relevant, since higher exposure to a drug is thought to increase toxicity, while a lower exposure may lead to pharmacokinetic resistance, resulting in a lower survival. For several TKIs exposure-response or exposure-toxicity relations have been found [11]. In the case of erlotinib and osimertinib, no definite target plasma concentrations have been described to optimize their efficacy [11]. Concerning toxicity, pharmacologic modelling has shown that osimertinib concentrations have a positive relationship with occurrence of skin rash and diarrhea, and increase in cardiac QTc time [14]. For erlotinib, there is evidence that its main toxicity (diarrhea and skin rash) is correlated with dosage and drug concentrations [15,16,17]. In addition to TKI exposure, the possibility of penetration of the blood-brain barrier is of interest as brain metastases are a frequent site of metastasis and progression in NSCLC. Of all EGFR TKIs, drug penetration across the blood-brain barrier is highest for osimertinib [18]. This explains the lower incidence of central nervous system (CNS) progression for osimertinib compared to other EGFR-TKIs [19].

Although tissue biopsy is still considered the gold standard in defining the histological diagnosis and enabling extensive molecular investigation, the possibilities of plasma investigations for cell-free DNA (cfDNA) from a blood sample are increasing [20,21]. Besides the benefit of being a less invasive procedure with negligible risks, the cfDNA is likely to represent the full spectrum of clonal variation in the cell-free tumor DNA (ctDNA) as opposed to tissue obtained from just one tumor lesion/region [22]. Detection of primary activating *EGFR* mutations and resistance mechanisms, e.g., p.T790M development in plasma, have shown to be adequate and effective for directing therapy [23].

Our study aimed to explore the predictive value of blood-based biomarkers including cfDNA plasma mutation detection and drug level monitoring in patients with *EGFR*-mutated NSCLC treated with a first or second line EGFR-TKI.

## 2. Results

Between March 2017 and May 2019, a total of 41 unique patients with *EGFR*-mutated NSCLC, treated with a 1st generation EGFR-TKI in first line or 3rd generation EGFR-TKI in second line for a *EGFR* p.T790M resistance mutation, were included. Five patients were enrolled twice, both at first line and second line treatment. Hence, 46 observed treatment lines were available. Minimal follow-up was six months. Median follow-up of patients still alive at data cut-off at December 1st 2019 was 14.9 months (range 6.4–34.0 months).

### 2.1. Baseline Characteristics

Baseline characteristics are presented in Table 1. In the total cohort of individual patients (*n* = 41), the median PFS in the first line cohort (13.6 months (*n* = 14, 95% confidence interval (CI) 3.2–23.9 months)) was comparable to the PFS in the second line cohort (11.5 months (*n* = 27, 95% CI 3.2–19.8, *p* = 0.768)), see Supplementary Data Figure S1.

At baseline, samples for NGS were available in all patients: tissue samples were available in 32 patients (78%) and plasma in 38 (93%) patients of the total population (*n* = 41). In 31 out of 38 plasma samples ctDNA was detected (82%). There was no significant difference in PFS in patients with or without detectable ctDNA at baseline (see Supplementary Data Figure S2). However, the patients without baseline *EGFR* mutations in plasma (*n* = 7) did not have any radiological progression events, compared to 22 events in patients with detectable *EGFR* mutations at baseline (*n* = 31). At data cut-off, five patients were still on treatment and two died of other reasons (one patient due to multi-organ failure with empyema after chest tube placement, the other patient suddenly passed away at home after sudden onset of dyspnea, presumably because of pulmonary embolism or a cardiac event, see also Supplementary Data Table S1). In one patient a *MAP2K1* mutation was found in absence of *EGFR* mutations in plasma at baseline, which was not detected in the tumor tissue, this patient was still on treatment at data cut-off. Treatment during study and best response are summarized in Table 2.

### 2.2. Presence of Concomitant Mutations (besides EGFR Primary Mutation and p.T790M)

At baseline, 26 patients harbored concomitant mutations (63%, *n* = 41), including *TP53*, *ERBB2*, *CTNB1*, *MTOR*, *CDKN2A*, *ARAF*, *PIK3CA*, *PTEN*, *MAP2K1*, and *APC* mutations. In 11 patients (27%) more than one concomitant mutation was detected and all of them included a *TP53* mutation. One patient had more than one concomitant mutation found in plasma, with a *PIK3CA* mutation besides *EGFR* and *TP53* mutations, and an additional *PTEN* mutation in tissue. There was no significant difference in PFS in patients with no, one or more concomitant mutations, see Supplementary Data Figure S3.

### 2.3. Presence of TP53 Mutations

A *TP53* mutation was detected in 23 patients (56%); seven times a *TP53* mutation was detected in plasma, compared to 22 in tissue. In one case, the *TP53* mutation in plasma did not correspond to the mutation in tissue at baseline, but did agree with the tissue mutation at radiologic progression. *TP53* mutations were most common in exon 7 and exon 5, the majority were missense mutations, see Table 3. In the total *EGFR* cohort, median PFS in patients without a *TP53* mutation at baseline (*n* = 10, 18.8 months, 95% CI 13.5–24.1) was not significantly longer than median PFS in the *TP53* mutated group (*n* = 23, 13.1 months, 95% CI 4.1–22.1, *p* = 0.068). In the second line cohort, the PFS was significantly shorter in thirteen patients with a *TP53* mutation at basel ine than in six wild-type patients (8.8 (95% CI 0.7–16.9) vs. 18.8 (95% CI 13.3–24.3) months, *p* = 0.017), see Supplementary Data Figure S4C. In one patient a new *TP53* mutation was detected in tumor tissue at time of progressive disease (PD). At baseline however, no tissue was available for this patient. No other new *TP53* mutations were demonstrated over the course of treatment.

### 2.4. Resistance Mechanisms

At radiologic progression, in 19 patients (83% out of 23 patients with available samples) at least one new concomitant mutation was detected, of which 12 (52%) had more than one concomitant mutation.

#### 2.4.1. First Line Cohort (1st Generation EGFR-TKI)

At data cut-off, 15 of the 19 patients had a PFS event (79%), while 13 showed radiological progression of disease (68%). Plasma samples for analysis at PD were available in 11 patients and tissue samples in seven of these patients. In six patients treated with erlotinib or gefinitib a *EGFR* p.T790M mutation was detected at PD; in two patients *EGFR* p.T790M was detected in plasma but not in tissue (including one missing tissue sample), two cases had *EGFR* p.T790M confirmed in both plasma and tissue, and in two other patients *EGFR* p.T790M was present in tissue but not in plasma. Two patients also developed an extra possible resistance mutation in tissue samples, a novel *CDKN2A* homozygote deletion and a novel *APC* mutation next to *EGFR* p.T790M.

#### 2.4.2. Second Line Cohort (Osimertinib)

At data cut-off, 18 of 27 patients had a PFS event, of whom 16 demonstrated radiological progression (59%). At PD, plasma samples were collected in all 16 patients with radiologic progression, and a new tumor biopsy was taken in 12 cases. In 14 patients different genetic alterations at PD compared to baseline were detected. These possible resistance mechanisms to osimertinib were divided into on-target and off-target resistance mechanisms. On-target mechanisms were observed in four patients, including three *EGFR* p.C797S mutations. In addition, 21 off target mutations were observed in 11 patients. In three patients, the tumor transformed to a small-cell lung cancer, all having a *TP53* and *PIK3CA* mutation at PD. Loss of *EGFR* p.T790M mutation was observed in nine patients, including all patients with a transformation to small-cell lung cancer. Other emerging mutations were *BRAF* p.V600E, *CDKN2A* homozygous deletions, *MET* amplifications, and *PTEN* mutations. In two patients, the resistance mutation was detected in plasma, as tissue was not available (*EGFR* p.C797S and *BRAF* p.V600E). An extra mutation was detected in two cases, which were not found in tissue (*PIK3CA* mutation and a *BRAF* mutation). In another patient, a *PIK3CA* mutation was detected in both plasma and tissue.

### 2.5. Plasma Conversion

#### 2.5.1. Plasma Conversion to cfDNA Negative for the Primary EGFR Mutation

**Figure 1:**
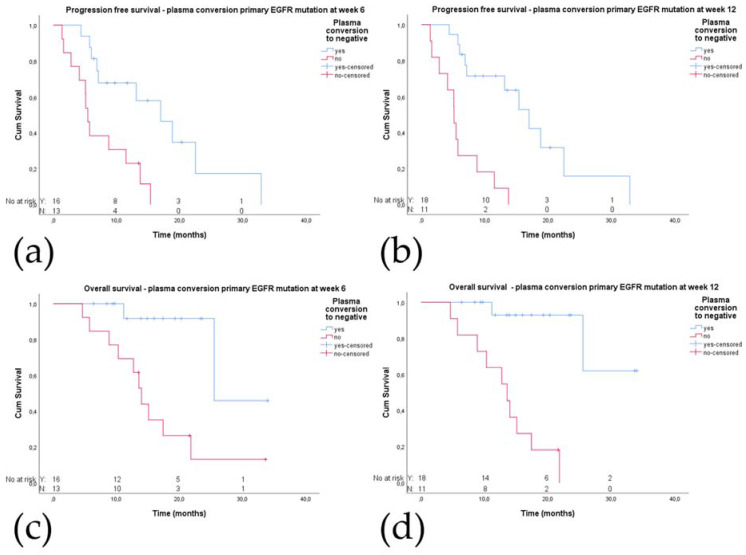
Plasma conversion of the primary epidermal growth factor receptor (EGFR) mutation in relation to progression-free survival (PFS) and overall survival (OS): (**a**) PFS and plasma conversion at week 6; (**b**) PFS and plasma conversion at week 12; (**c**) OS and plasma conversion at week 6; (**d**) OS and plasma conversion at week 12.

Plasma conversion status was evaluable in 29 patients at week 6 and 12 after start of treatment. Sixteen and 18 patients had complete plasma conversion at week 6 and 12 respectively. Patients with complete plasma conversion had a significantly longer PFS compared to patients without complete plasma conversion at either week 6 (17.0 (95% CI 9.7–24.2) vs. 5.5 (95% CI 4.7–6.2) months, *p* = 0.002; Figure 1a), and week 12 (17.0 (95% CI 11.7–22.3) vs. 5.1 (95% CI 3.7–6.6) months, *p* < 0.001; Figure 1b). Illustrative, both patients who reached complete plasma conversion at week 12 already had an almost complete conversion of the primary *EGFR* mutation at week 6 (−80 and −97% respectively). These significant differences in PFS in patients with complete plasma conversion were also present in separate analyses of the treatment cohorts, see Supplementary Data Table S2.

In addition, OS was significantly longer in patients with plasma conversion at week 6 compared to patients with continuous detection of the primary *EGFR* mutation (25.5 (95% CI could not be calculated) vs. 14.0 (95% CI 12.0–16.0) months, *p* = 0.003). This difference in OS was also present at week 12 (NR vs. 13.6 (95% CI 9.6–17.5) months, *p <* 0.001; Figure 1c,d).

**Figure 2:**
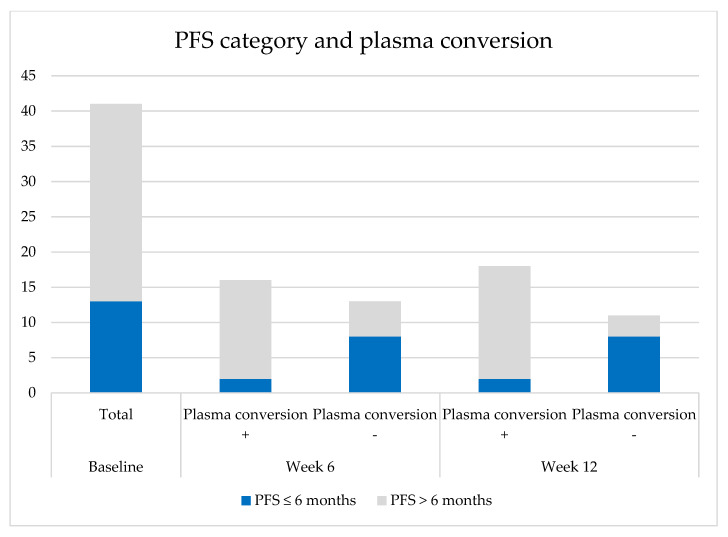
PFS category distribution and plasma conversion in time.

Twelve (75%) of the patients with complete plasma conversion at week 6 had a partial response (PR) and four (25%) had stable disease (SD) as best radiologic response. In the case of a lack of complete plasma conversion, only five patients (39%) had a PR, six (46%) had SD, and two (15%) PD as best response (chi square *p* = 0.064). The share of patients with a short and long PFS in relation to plasma conversion is depicted in Figure 2.

#### 2.5.2. Plasma Conversion to cfDNA Negative for the EGFR p.T790M Mutation

In the second line cohort (osimertinib), *EGFR* p.T790M was evaluated in plasma. Ten patients without plasma conversion of the primary mutation, but with loss of *EGFR* p.T790M in plasma at week 6 had a significant shorter PFS of 5.1 months (95% CI 4.6–5.7) compared to 11 patients with plasma conversion for both the primary *EGFR* and the p.T790M mutation with a PFS of 18.8 months (95% CI 7.5–30.1, *p* = 0.012), see Supplementary Data Figure S5.

#### 2.5.3. Plasma Mutation Levels during Treatment

**Figure 3:**
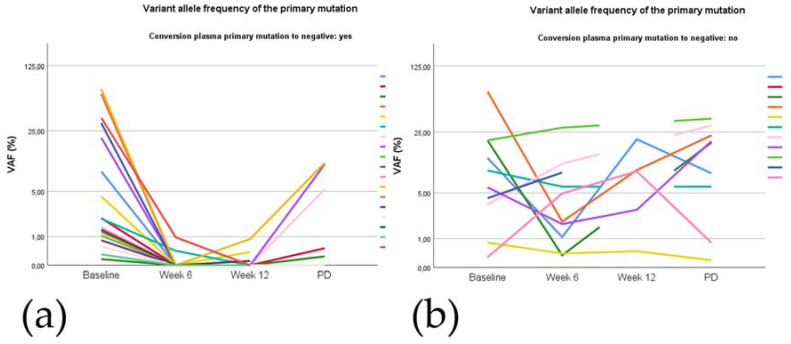
Variant allele frequency (VAF) of the primary EGFR mutation in time, by plasma conversion status. (**a**) Patients with complete plasma conversion; (**b**) patients without complete plasma conversion.

There was no correlation between baseline and progression levels of the primary *EGFR* mutation (i.e., variant allele frequency) or cfDNA concentrations in plasma. In most patients in the second line cohort, there was no detectable *EGFR* p.T790M at progression in contrast to baseline, while the primary *EGFR* mutation was detectable in the majority of patients, see Supplementary Data Figure S6. The allele fraction of the primary EGFR mutation was widely variable among patients with and without plasma conversion, as shown in Figure 3.

### 2.6. Short Responders

Thirteen patients (32%) met the definition of being a short responder (PFS < 6 months). This group included relatively fewer patients with an exon 19 primary *EGFR* mutation (7 cases, 54%) than in the group with a response of more than 6 months (21 out of 28 cases, 75%, NS). The 2 current smokers at presentation were both short responders, of the 15 never smokers only 2 (13%) were short responders (chi square *p* = 0.022). Of the patients with evaluable plasma and tissue samples (*n* = 33), all short responders had a *TP53* mutation at baseline compared to 58% in the groups with a PFS of more than 6 months (chi square *p* = 0.032). Of the five patients with three other concomitant mutations, besides *EGFR* mutations, three (60%) were short responders, while eleven of the 15 patients without other concomitant mutations (73%) had a response >6 months (NS). Of the patients evaluable for PFS category and plasma conversion at week 6 (*n* = 29), only two patients (13%) reached complete plasma conversion among the short responders, opposite to 88% for patients with PFS > 6 months (chi square *p* = 0.009). The two short responders who did reach complete plasma conversion were a patient with cerebral oligoprogression on erlotinib, and a patient with SCLC transformation during osimertinib as second line treatment.

### 2.7. Plasma Drug Concentrations

**Figure 4:**
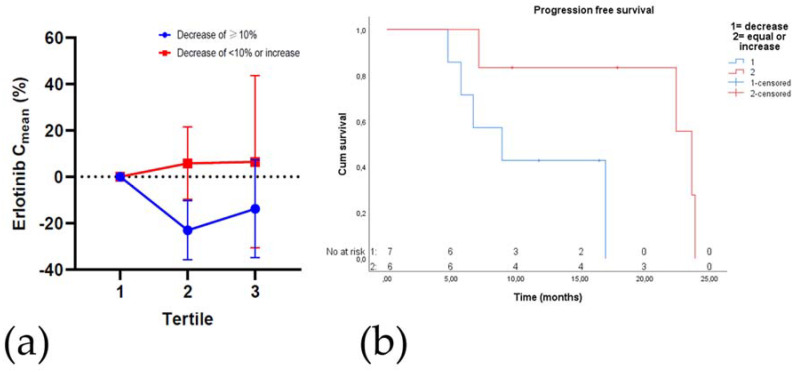
(**a**) Relative change in erlotinib C_mean_ during treatment. Treatment period is divided in tertiles; (**b**) progression-free survival based on the erlotinib C_mean_ in the second tertile compared to the first tertile. The first (red-line) group has a decrease of less than 10% or an increase in C_mean_. The second (blue-line) group has a decrease of at least 10% in erlotinib C_mean_. C_mean_ = mean plasma concentration.

In 15 patients treated with erlotinib and 22 treated with osimertinib, a total of 258 samples were drawn for pharmacokinetic analysis. Interestingly, a decrease in erlotinib C_mean_ in the second tertile was correlated with a significantly shorter PFS (median PFS 8.9 (95% CI 3.2–14.6) vs. 23.6 (21.7–25.6) months; *n* = 13; log-rank *p* = 0.037), see Figure 4. Additionally, patients treated with erlotinib who experienced a decreased C_mean_ two months prior to PD, compared to C_mean_ until six weeks after treatment initiation, had a significantly lower PFS (4.7 (95% CI could not be calculated) vs. 7.1 (95% CI 6.4–7.8) months; *n* = 5; *p* = 0.039). The average time to second tertile in patients with a decreased C_mean_ was 20 weeks. Notably, patients treated with erlotinib in whom the C_mean_ decreased in the second tertile compared to the first tertile of treatment, had a significantly shorter time until severe toxicity occurred (median 11.8 (95% CI 10.6–12.9) vs. 23.9 (95% CI could not be calculated) months; *n* = 13; *p* = 0.031). Dose reductions were, although non-significant, more frequent in this group with decreased C_mean_ (67% vs. 29%; *p* = 0.17).

Median C_mean_ during treatment was 1085 ng/mL for erlotinib and 190 ng/mL for osimertinib. Table 4 presents an overview of the results of the Kaplan-Meier analysis with the log-rank test to correlate (changes in) C_mean_ during treatment with PFS. The correlations with erlotinib and PFS were not seen in patients treated with osimertinib (all log rank *p* > 0.05; Table 4).

#### Toxicity

All reported TKI-related toxicities are presented in Table 5. Fourteen patients experienced severe toxicity; four (15%) occurred in the osimertinib-treated patients, and ten (56%) in the erlotinib-treated patients.

The occurrence of severe toxicity was not correlated with C_mean_ divided by median for either drug (Table 4). However, all patients who experienced osimertinib-related severe toxicity had a C_mean_ above the median C_mean_ (33 vs. 0%, chi square *p* = 0.062).

### 2.8. Brain Metastasis

At start of treatment, in the first line cohort, the one patient on gefitinib did not have CNS disease, but three patients (17%, *n* = 18) treated with erlotinib and five patients (19%, *n* = 27) in the second line cohort had intracerebral metastasis. There was no significant difference in *TP53* mutational status in patients with or without baseline intracerebral metastasis (*p* = 0.399). Six patients were evaluable for plasma conversion status, and all six had undetectable primary mutation after 6 weeks of treatment vs. 46% who had no CNS disease at start (chi square *p* = 0.017). If patients had CNS disease at baseline, those treated with erlotinib had a significantly lower PFS compared to patients without CNS disease at baseline: 6.7 (95% CI 5.2–8.2) vs. 17.0 (95% CI 5.2–28.2) months, *n* = 18; *p* = 0.032.

From the eight patients with CNS disease at start of treatment, one in each treatment line had intracerebral PD. In total three patients in each cohort had (new) intracerebral disease as site of progression (17% and 11% for erlotinib and osimertinib respectively; chi square *p* = 0.591). Comparing the patients with and without CNS as site of PD, mean TKI concentrations in plasma did not significantly differ for neither erlotinib (1390 vs. 1016 ng/mL; *p* = 0.461) nor osimertinib (206 vs. 188 ng/mL; *p* = 0.321). There was no significant difference in presence of *TP53* mutations at baseline or C_mean_ in the patients with or without CNS progressive disease (see Supplementary Data Table S3). Five of the six patients with CNS PD (83%) had concomitant *TP53* mutations at baseline vs. 68% who had no or stable CNS disease (chi square *p* = 0.118). In four patients the primary mutation was detectable in plasma at baseline, but all converted to undetectable after 6 weeks (100% vs. 50%; chi square *p* = 0.060).

### 2.9. Multivariate Analysis

Multivariate analysis with Cox regression was performed for complete plasma conversion of the primary EGFR mutation at week 6 and presence of the *TP53* mutation at baseline. In the total cohort, complete plasma conversion was significantly correlated with PFS; HR 0.23, 95% CI 0.07–0.77, *p* = 0.017. However, *TP53* was not significantly correlated with PFS; HR 2.71, 95% CI 0.72–10.23, *p* = 0.143.

## 3. Discussion

The emergence of EGFR-TKIs has led to an astounding improvement in survival of patients with *EGFR*-mutated lung cancer. However, a minority of patients do not benefit and have no or just a short-living response. The use of liquid biopsies is promising, as it is a minimal invasive procedure, which is easily performed in clinical practice during routine outpatient clinic visits. Therefore, in this prospective study, both cfDNA and TKI drug concentrations were monitored during EGFR-TKI treatment to identify predictive markers to be used in clinical practice.

First of all, this study shows that absence of complete plasma conversion of the primary *EGFR* mutation at either week 6 or 12 was associated with a significantly shorter PFS and OS. This detrimental effect was demonstrated in the total *EGFR* cohort, as well as in the treatment cohorts. These results therefore do not only support the finding that disappearance of the primary *EGFR* mutation in plasma is associated with a better outcome in first line [24,25], but also confirm its predictive value in the second line for osimertinib-treated patients with *EGFR* p.T790M [26]. The concept of losing detectable *EGFR* mutations in plasma in association with better outcome was recently also demonstrated at other centers [26], but often with less sensitive techniques (e.g., Cobas^®^) than our NGS panel [25,27], or cross-sectional at a single time point rather than sequentially analyzed as a change in time [28,29]. In our second line cohort, isolated *EGFR* p.T790M loss in plasma at week 6 was associated with decreased PFS (5.1 vs. 18.8 months). This has been previously observed and may be explained by the emergence of a pre-existing resistant sub-clonal population [30,31].

Second, this study shows strong signs of the detrimental effect of a baseline concomitant *TP53* mutation, despite the limited size of the study population. In patients treated with osimertinib, cases who harbored a *TP53* mutation at baseline had a significantly shorter PFS than *TP53* wild-type patients (8.8 vs. 18.8 months). *TP53* mutations were also more common among the short responders in the total cohort. As one of the main functions of *TP53* is the prevention of accumulation of genetic defects, a dysfunctional p53 could result in increased genomic instability and faster development of resistance mechanisms [32]. *TP53* mutations in our cohort, as in general, do not seem to develop during treatment, for they are described to occur early in oncogenesis [33]. Worse outcome associated with *EGFR*-and *TP53*-mutated NSCLC has been well established and our findings are in line with these results [5,34]. Besides *TP53* mutations, other concomitant mutations were also found. There was a relative increase in concomitant genetic aberrations between baseline and radiologic progression (more than one concomitant mutation in 27% and later 52% of samples). However, because of limited coverage of the cfDNA panel used, it was not possible to prove a relation between multiple concomitant mutations or *TP53* mutational status and PFS based on plasma analysis, although this association has been previously described in tissue [5,6]. Next, the *Rb1* gene, associated with SCLC histological transformation, was not covered by the NGS panel. Nevertheless, in this study the patients with SCLC transformation had a distinct mutational profile, which included *TP53* and *PIK3CA*, already at baseline, suggesting *PIK3CA* might also play a role in this dedifferentiation [35].

Third, this study found evidence for a relationship between erlotinib C_mean_ during treatment and PFS. Patients with a decrease of C_mean_ of 10% or more 2 months prior to PD compared to first 6 weeks had a significantly shorter median PFS (4.7 vs. 7.1 months). This result should be interpreted with caution though, since only five patients were included in this analysis. Additionally, patients with a decrease of C_mean_ of 10% or more in the second tertile compared to the first tertile had an significantly lower PFS compared to patients with an equal or increased C_mean_ (8.9 vs. 23.6 months). This decrease could be caused by multiple factors, i.e., dose reductions due to intolerable toxicity, increase in smoking behavior, decrease in therapy adherence, or use of concomitant interacting medication [13]. The contribution of dose reductions is illustrated by a higher prevalence of dose reductions and a significant shorter median time to severe toxicity in the C_mean_ decreased group. Before extrapolation to clinical practice, these results should be validated in a larger cohort.

The same percentage of patients had CNS disease at start of therapy in both cohorts. However, when CNS disease progression occurred, this percentage was almost twice as high in the first line cohort compared to the second line cohort where all patients were treated with osimertinib (19% vs. 10%). These results are in line with the FLAURA study results, that conclude that patients with *EGFR*-mutated NSCLC and CNS disease should preferably be treated with osimertinib [3].

Additional to the advantage of being a non-invasive procedure, the difference in the number of plasma cfDNA samples vs. tissue samples at baseline (92% vs. 78%) and at progression (82% vs. 54%) in our study reflects the feasibility of blood sampling. Moreover, plasma sampling reflects the total of genomic aberrations and heterogeneity of the disease. When in the near future reliable detection of translocations and amplifications in cfDNA is possible, plasma diagnostics could replace tissue-based investigation in current daily practice in some cases.

Limitations of this work are, first the limited number of included patients which limits the power for some of the analyses. Moreover, ctDNA could not be detected in all patient samples (ctDNA detected in 31 of 38 patients). In theory, this limits the sensitivity of mutation detection in plasma. However, patients without detectable ctDNA at baseline had a lower rate of radiological progression during follow-up compared to patients with detectable ctDNA baseline. The association between detectable ctDNA at baseline and shorter PFS and OS was previously reported by Buder et al. [26]. This could be explained by a lower tumor load or intrathoracic/-cerebral localization which is associated with less shedding of cfDNA by the tumor [20,36]. Additionally, the coverage of the mutation spectrum in the Oncomine lung cfDNA panel is a limitation in the detection of the aberrations, specifically in the *TP53* gene in our study. At this point, the NGS panel is more extensive for tissue than for plasma, which mostly explains the difference in detection of *TP53* mutations between tissue and plasma, see Supplementary Data Table S4. Other reasons could include limited input of DNA, or absence of cfDNA in plasma, excreted by the tumor. As false negative results are an important limitation, the occurrence of “false” positive mutations derived from cfDNA is also a realistic limitation. Plasma cfDNA analysis cannot make a distinction between mutations originating from the tumor or somatic mutations from nonmalignant peripheral blood cells, known as clonal hematopoiesis [37,38]. One possibility to overcome this limitation is to concurrently sequence peripheral blood cells [37]. In our study, *TP53* mutations found in plasma were matched with tissue samples. One *TP53* mutation detected in plasma did not match the *TP53* mutation observed in tumor tissue and was thus labeled as clonal hematopoiesis. When interpreting plasma cfDNA analysis clonal hematopoiesis should be considered in order to prevent misdiagnosis of malignancies. However, when plasma cfDNA analysis is used as a predictive and follow-up tool in EGFR-mutated NSCLC, we believe this is less relevant as mutations can be matched to tumor tissue.

This study shows multiple predictive features in plasma to identify patients with less benefit of EGFR-TKI treatment. The absence of complete plasma conversion of the primary EGFR mutation is associated with significant shorter PFS and OS, as well as *EGFR* p.T790M loss alone in plasma in patients treated with osimertinib in second line. Decrease of C_mean_ in time is also associated with shorter PFS in erlotinib treatment. The presence of TP53 mutations at baseline was associated with a shorter PFS.

After validation of these results in a larger and independent cohort, implementation to clinical practice seems practically possible when at start of EGFR-TKI treatment, cfDNA and PK samples are taken. At baseline, patients with *TP53* mutations on second line osimertinib treatment could already be considered in danger of progressing early. If cfDNA levels of the primary mutation are still detectable after 6 weeks of treatment, patients are at high risk of a short PFS, which could guide the treating clinician to more close follow-up or consideration of more extensive treatment options. For erlotinib-treated patients, monitoring of plasma drug concentrations is feasible. When either a 10% or more decrease in erlotinib C_mean_ occurs after 20 weeks, or when treatment conditions change (for example when C_mean_ decreases because a dose reduction is necessary) this could indicate to a shorter PFS as well. Altogether, the treating physician could use plasma predictive features to consider if closer follow-up or more extensive treatment might be necessary to personalize treatment of patients with *EGFR*-mutated NSCLC.

## 4. Materials and Methods

The START-TKI study is a prospective, observational multicenter study in which additional blood samples are drawn to standard outpatient visits in patients treated with a TKI for driver mutation driven NSCLC. The study was approved by the medical ethical committee of the Erasmus MC, Rotterdam, The Netherlands (MEC 16-643), and initiated at two sites; Erasmus MC, Rotterdam and Amphia hospital, Breda, The Netherlands. Written informed consent was obtained from all patients prior to study enrollment.

### 4.1. Study Population

Patients were eligible for the *EGFR* cohort when harboring an activating *EGFR* mutation for which first or second line EGFR-TKI treatment was initiated.

### 4.2. Blood Collection and Processing

Blood samples were collected at baseline, prior to start of EGFR-TKI, and at every outpatient clinic visit following standard of care (i.e., every six weeks) until progressive disease (PD) or end of treatment due to toxicity. For cfDNA isolation, two Cellsave preservative 10mL vacutainer tubes (CellSearch, Menarini Silicon Biosystems, Castel Maggiore, Italy) were drawn. The cfDNA handling has been previously described [39]. Additionally, a 4.0 mL lithium-heparin tube was taken for pharmacokinetic analysis.

#### 4.2.1. Next Generation Sequencing (NGS)

NGS was performed at baseline, at week 6 and 12 and at PD on isolated cfDNA from plasma and tissue if available [39,40]. NGS was performed by semiconductor sequencing with the Ion S5 System (Thermo Fisher Scientific) with the supplier’s materials and protocol. Libraries from tissue DNA were prepared with a custom-made primer panel. cfDNA library preparation was performed using the Oncomine Lung cfDNA Assay v1 (Thermo Fisher Scientific), coverage is available in the Supplementary Data Table S5. Standard depth of sequencing was 25.000× on average. Our NGS process including the customized tissue panel has been described in more detail earlier [39,40]. NGS on tumor tissue at diagnosis was done as part of routine clinical care.

#### 4.2.2. Plasma Drug Concentrations

Patients were asked to delay the intake of TKI tablet until after blood withdrawal. All samples were analyzed with validated liquid chromatography-tandem mass spectrometric assays [41,42]. Additionally, samples from patients included in this study who also participated in a previous pharmacokinetic study with erlotinib were integrated [43]. Time between last intake and blood withdrawal was calculated with the patient reporting time of last intake. Concentrations from samples collected after the label’s time to reach maximum concentration (T_max_; *c.q.* 4 h for erlotinib and 6 h for osimertinib) were extrapolated to the trough concentration at 24 h (C_24 h_) after drug intake with the following equation:

$$C_{24\;h}=C_{\mathrm{sample}}*e^{\left(-{\;T}_{\mathrm{to}\;24h\;}*\;24\;*\;\frac{0.693}{T_{\mathrm{half}}}\right)}$$

C_24 h_ data for each patient were used to calculate the mean plasma concentration (C_mean_). The C_mean_ of total treatment duration was analyzed with Cox regression to study the relation between drug exposure and PFS. The C_mean_ for the first six weeks of treatment and two months before PD were calculated. Hypothetically, a decrease in C_mean_ during treatment would be an explanation for PD. To further investigate whether changes in C_mean_ could have an influence on PFS, C_mean_ was calculated for every third of total treatment duration per patient (i.e., tertiles). Changes in C_mean_ during treatment were subtracted from these data. To compare the forthcoming changes in C_mean_ between tertiles, two groups have to be defined; one in which C_mean_ decreases and one in which no change or even increase in C_mean_ occurs. Additionally, C_mean_ was calculated for time until the occurrence of severe toxicity.

### 4.3. Toxicity

Toxicity was scored by the treating physician according to the Common Terminology Criteria for Adverse Events (CTCAE) grading system version 4.03 [44]. Severe toxicity was defined as CTCAE grade ≥ 3 or hospital admission, dose reduction, and treatment discontinuation or stop because of TKI-related toxicity.

### 4.4. Brain Metastasis

Patients who had CNS metastasis at baseline and at PD were identified. Mean TKI concentrations were compared for CNS metastasis at PD. Also the presence of *TP53* mutations and complete plasma conversions was compared between patients with and without CNS metastasis.

### 4.5. Objectives

Main objectives were exploration of the predictive value of the presence of concomitant mutations in cfDNA at baseline and TKI plasma trough concentrations (C_min_) during treatment for PFS. In addition, the associations between change in plasma mutation levels over time and PFS and OS were analyzed. Plasma and tissue mutations were analyzed and compared between different time points.

Additionally, the correlation between TKI plasma trough concentration and occurrence of severe toxicity and the relationship between brain metastasis and pharmacokinetic parameters was explored.

### 4.6. Statistical Analysis

PFS was defined as time from start TKI until radiologic progression or death, OS as time from start TKI until death. Short responders were defined as a PFS < 6 months. We defined plasma conversion as the shift from detectable to undetectable mutation status in plasma. Patients who were enrolled twice (both in first and second line treatment) were included for separate analyses in treatment cohorts (1st line cohort and 2nd line cohort). For analysis of the total population, from these patients only data from the second line cohort were used.

The relationship between PFS and presence of concomitant mutations, *TP53* mutations specifically, plasma mutation conversion and changes in C_mean_ was explored by the log-rank test on Kaplan Meier survival analysis. Kaplan Meier analysis was used for estimation of median survival times and 95% confidence intervals. In case of a limited number of events at data cut off the 95% confidence interval could not be calculated. Differences between groups were compared with Pearson chi-square tests (i.e., for prevalences) or the T-test (i.e., for mean concentrations). To correlate the influence of multiple variables on PFS, Cox regression was performed on the variables that were significantly (*p* < 0.05) correlated with PFS in univariate analysis in the total *EGFR* cohort. IBM SPSS Statistics version 25 was used for all analyses.

## 5. Conclusions

Not all patients treated with EGFR-TKIs benefit from a durable response, but predictive markers to identify these short responders are lacking. This prospective blood-based biomarker study, START-TKI, in *EGFR*-mutated NSCLC patients reports poor predictive markers based on cfDNA and TKI drug concentrations during EGFR-TKI treatment which have potential to be used in clinical practice in the future. Absence of complete plasma conversion of the primary *EGFR* mutation at week 6 and 12 was correlated with significantly shorter PFS and OS. Concomitant *TP53* mutations at baseline also showed signs of detrimental outcome. Patients treated with erlotinib who had a decrease in mean plasma drug concentration of 10% or more during treatment had worse PFS, but in a small cohort. Validation in a larger cohort is preferred. Implementation of these plasma predictive features could aid a physician to consider for which EGFR-TKI-treated patients closer follow up or more extensive treatment might be necessary.

## Figures and Tables

**Table 1:** Baseline characteristics.

Baseline Patient Characteristics	*n* = 41
Gender	
Male	18 (44%)
Female	23 (56%)
Age (median, range)	62 (42–83)
Ethnicity	
Caucasian	36 (88%)
Asian	4 (10%)
Unknown	1 (2%)
Smoking status	
Current	2 (5%)
Former	24 (58%)
Never	15 (37%)
Former pack years (median, range)	6 (0–40)
0	15 (37%)
1–15	11 (27%)
15–30	9 (22%)
≥30	1 (2%)
Unknown	5 (12%)
Histology	
NSCLC; adenocarcinoma	39 (95%)
NSCLC NOS	1 (2.5%)
Unknown	1 (2.5%)
Type of primary *EGFR* mutation	
Exon 19	28 (68%)
Deletion	23 (56%)
Deletion-insertion	4 (9.5%)
Other (VUS)	1 (2.5%)
Exon 21	13 (32%)
p.L858R	12 (29.5%)
Other	1 (2.5%)
Exon 20 concomitant mutation	28 (68%)
p.T790M	27 (66%)
Exon 19 + exon 20	1 (2.5%)
Plasma available at baseline	38 (93%)
Tissue available at baseline	32 (78%)

**Table 2:** Treatment lines during study.

Available Treatment Lines during Study	Total Cohort * (*n* = 41)	1st Line Cohort (*n* = 19)	2nd Line Cohort (*n* = 27)
Best response on treatment			
PR	25 (61%)	13 (68%)	15 (56%)
SD	12 (29%)	4 (21%)	10 (37%)
PD	3 (7%)	2 (11%)	1 (3.5%)
Unknown	1 (3%)	0 (0%)	1 (3.5%)
Progression or death event	28 (68%)	15 (79%)	18 (67%)
Radiological progression	24 (59%)	13 (68%)	16 (59%)
Death without radiological progression	4 (10%)	2 (11%)	2 (28%)
Progression free survival category			
≤6 months	13 (32%)	4 (21%)	9 (33%)
>6 months	28 (68%)	15 (79%)	18 (67%)

* To prevent bias, we only included the most recent treatment lines of the 41 individual patients, 5 treatment lines from the 1st line cohort were excluded in analyses of the total *EGFR* cohort. Abbreviations: PR = partial response, SD = stable disease, PD = progressive disease.

**Table 3:** *TP53* status at baseline.

Baseline *TP53* mutation present (*n* = 41)	23 (56%)
Present in plasma (*n* = 23)	6 (26%)
Present in tissue (*n* = 23)	22 (96%)
No plasma sample baseline	2 (9%)
No ctDNA baseline	4 (17%)
Not covered by cfDNA panel	6 (26%)
Covered by cfDNA panel	4 (17%)
Location of mutation	
Exon 5	6 (26%)
Exon 6	3 (13%)
Exon 7	8 (35%)
Exon 8	2 (9%)
Other	4 (17%)
Type of mutation	
Missense	15 (65%)
Nonsense	1 (5%)
Other	7 (30%)

**Table 4:** Kaplan-Meier analysis to correlate (changes in) C_mean_ during treatment with PFS and toxicity.

Variable	Event	Erlotinib		Osimertinib	
		*n* (Events)	Log-Rank *p*-Value	*n* (Events)	Log-Rank *p*-Value
C_mean_ of total treatment ^†^	PFS	15 (11)	0.517	22 (13)	0.631
C_mean_ first six weeks ^†^	PFS	12 (8)	0.197	20 (11)	0.972
C_mean_ two months before PD ^†^	PFS	7 (7)	0.779	15 (9)	0.221
C_mean_ two months before PD/C_mean_ first six weeks ^‡^	PFS	5 (5)	0.039*	7 (7)	0.561
C_mean_ third tertile/C_mean_ first tertile ^‡^	PFS	8 (8)	0.855	17 (10)	0.821
C_mean_ second tertile/C_mean_ first tertile ^‡^	PFS	13 (9)	0.037*	17 (10)	0.169
C_mean_ third tertile/C_mean_ second tertile ^‡^	PFS	9 (9)	0.415	15 (8)	0.517
C_mean_ until severe toxicity or end of study ^†^	Tox	15 (10)	0.430	22 (3)	0.134
C_mean_ third tertile/C_mean_ first tertile ^‡^	Tox	8 (4)	0.433	17 (2)	0.705
C_mean_ second tertile/C_mean_ first tertile ^‡^	Tox	13 (8)	0.031 *	17 (3)	0.460
C_mean_ third tertile/C_mean_ second tertile ^‡^	Tox	9 (5)	0.786	15 (2)	0.564

^†^ = Variable group deviated by median; ^‡^ = variable group deviated by a decrease of 10% or more; * = statistically significant (*p* < 0.05). Abbreviations: C_mean_ = mean plasma drug concentration at 24 h; PFS = progression-free survival; *n* = number of patients; PD = progressive disease.

**Table 5:** Toxicity.

Erlotinib (*n* = 18)	
Severe toxicity	10 (56%)
CTCAE grade ≥ 3	5 ^†^ (28%)
Hospital admission	2 (11%)
Dose reduction	8 (44%)
TKI interruption/discontinuation	2 (11%)
Erlotinib specific toxicity (all grade)	
Rash	12 (72%)
Alopecia	8 (44%)
Diarrhea	6 (33%)
Sicca (dry eyes, mouth and/or skin)	6 (33%)
Paronychia	5 (28%)
Hand-Foot Syndrome	1 (6%)
Hypertrichosis	1 (6%)
**Osimertinib (*n* = 27)**	
Severe toxicity	4 (15%)
CTCAE grade ≥ 3	4 * (15%)
Hospital admission	2 (7%)
Reduction	2 (7%)
TKI interruption/discontinuation	3 (11%)
Osimertinib specific toxicity (all grade)	
CK elevation	6 (22%)
Paronychia	5 (19%)
Diarrhea	4 (15%)
Dry skin	4 (15%)
QTc prolongation	4 ^‡^ (17%)
Pneumonitis	3 (11%)
Rash	2 (7%)

Abbreviations: n = number of patients; CTCAE = Common Terminology Criteria for Adverse Events. ^†^ = 1 rash, 1 nausea and vomitus, 1 kidney failure, 1 ALAT increase and 1 hypokalemia and pericarditis. * = 1 pneumonitis, 1 pneumonitis and skin infection and 1 CK elevation. ^‡^ = calculated for patients which had baseline and follow-up electro cardiograms (n = 23).

## Supplementary Materials

The following are available online at https://www.mdpi.com/2072-6694/12/11/3179/s1. Table S1: Causes of death events in cases without radiologic progression. Table S2: PFS and plasma conversion at week 6 and 12 in treatment cohorts. Table S3: C_mean_ in patients with CNS progressive disease. Table S4: Coverage discrepancies of the detected mutations in our study. Table S5: Coverage of the Oncomine Lung cfDNA assay v1. Figure S1: PFS in the total *EGFR* cohort according to treatment line. Figure S2: PFS in presence or absence of detectable ctDNA. Figure S3: PFS in patients with concomitant mutations. Figure S4: PFS and *TP53* mutational status. Figure S5: PFS and plasma conversion in the second line cohort. Figure S6: Plasma mutation levels in the second line cohort.
