# Supplementary material for: Plasma Predictive Features in Treating EGFR-Mutated Non-Small Cell Lung Cancer
Source: Cancers (Basel). 2020 Oct 29;12(11):3179. doi: 10.3390/cancers12113179 (PMC7692448; doi:10.3390/cancers12113179)
Supplement: Supplementary file 1 [file cancers-12-03179-s001.docx]

Supplementary Materials

Plasma Predictive Features in Treating EGFR-Mutated Non-Small Cell Lung Cancer

Christi M.J. Steendam, G.D. Marijn Veerman, Melinda A. Pruis, Peggy Atmodimedjo, Marthe S. Paats, Cor van der Leest, Jan H. von der Thüsen, David C.Y. Yick, Esther Oomen-de Hoop, Stijn L.W. Koolen, Winand N.M. Dinjens, Ron H.N. van Schaik, Ron H.J. Mathijssen, Joachim G.J.V. Aerts, Hendrikus Jan Dubbink and Anne-Marie C. Dingemans

**Table S1.** Causes of death events in cases without radiologic progression:

1. multi-organ failure associated with empyema after chest tube placement
2. hypoxemia in pulmonary embolism
3. hypoxemia due to pneumonitis (probably osimertinib related)
4. sudden onset of dyspnea at home, presumably because of pulmonary embolism or a cardiac event

**Table S2A.** PFS and plasma conversion at week 6 (treatment cohorts).

| Cohort | Plasma conversion | n/events | Median PFS (months) | 95% CI | Log rank *p*= |
| --- | --- | --- | --- | --- | --- |
| First line | Yes | 6/5 | 7.1 | 0.0-14.8 | 0.014 |
|  | No | 1/1 | 1.4 | NA |  |
| Second line | Yes | 11/6 | 18.8 | 7.5-30.1 | 0.011 |
|  | No | 12/11 | 5.5 | 4.4-6.5 |  |

**Table S2B.** PFS and plasma conversion at week 12 (treatment cohorts).

| Cohort | Plasma conversion | n/events | Median PFS (months) | 95% CI | Log rank *p*= |
| --- | --- | --- | --- | --- | --- |
| First line | Yes | 6/5 | 7.1 | 0.0-14.8 | 0.014 |
|  | No | 1/1 | 1.4 | NA |  |
| Second line | Yes | 11/6 | 15.4 | 9.3-21.5 | 0.000 |
|  | No | 12/11 | 5.1 | 4.6-5.7 |  |

**Table S3.** PK: C_mean_ in patients with CNS progressive disease.

Erlotinib 1390 vs. 1015 ng/mL; p = 0.461

Osimertinib 230 vs. 188 ng/mL; p = 0.097

**Table S4.** Coverage discrepancies of the detected mutations in our study.

| **Mutation** | **Covered by plasma panel** | **Covered by tissue panel** |
| --- | --- | --- |
| APC c.4399_4400dupCC; p.K1468Lfs* | No | Yes |
| ARAF c.558-1G>A;p.? VUS | No | Yes |
| BRAF c.1799T>A; p.V600E | Yes | Yes |
| BRAF p.469A | Yes | Yes |
| CDKN2A c.159G>A; p.M53I | No | Yes |
| CDKN2A c.163G>T; p.G55C | No | Yes |
| CDKN2A c.250G>A; p.D84N VUS | No | Yes |
| CDKN2A c.355G>T; p.E119* | No | Yes |
| CDKN2A homozygote deletie | No | Yes |
| CTNNB1 c.94G>T; p.D32Y | No | Yes |
| CTNNB1 c.110C>T; p.S37F | No | Yes |
| EGFR c.2170G>A; p.G724S | No | Yes |
| EGFR p.C797S (c.2389T>A, in CIS) | Yes | Yes |
| EGFR p.C797S (c.2390G>C, in CIS) | Yes | Yes |
| EGFR p.V769M, c.2305G>A | Yes | Yes |
| ERBB2 c.1963A>G, p.(Ile655Val) | No | Yes |
| ERBB2 c.2066G>A, p.(Arg686), | No | Yes |
| MAP2K1 p.E203K | Yes | Yes |
| MET amplificatie | No | Yes |
| MTOR c.7291C>A; p.L2431M | No | Yes |
| NTRK1 amplificatie | No | Yes |
| PIK3CA c.1636C>G; p.Q546E | Yes | Yes |
| PIK3CA c.3145G>C; p.G1049R | Yes | Yes |
| PIk3CA p.E542K | Yes | Yes |
| PIK3CA p.E545K | Yes | Yes |
| PIK3CA p.E545Q | Yes | Yes |
| PTEN c.388_400del; p.R130* | No | Yes |
| PTEN p.L320S, c.959T>C | No | Yes |
| PTEN c.131G>A; p.G44D | No | Yes |
| TP53 c.243_244dup; p.P82Hfs*42 | No | Yes |
| TP53 c.536A>G;p.H179R | Yes | Yes |
| TP53 c.395A>T; p.K132M | No | Yes |
| TP53 c.469G>T; p.V157F | Yes | Yes |
| TP53 c.673-1G>T; p.? | Yes | Yes |
| TP53 c.713G>T; p.C238F | Yes | Yes |
| TP53 c.733G>A; p.G245S | Yes | Yes |
| TP53 c.797G>T; p.G266V | Yes | Yes |
| TP53 c.892G>T; p.E298* | No | Yes |
| TP53 c.646G>A; p.V216M | Yes | Yes |
| TP53 c.97-1G>T, p.? | No | Yes |
| TP53 c.339_341del;p.F113del | No | Yes |
| TP53 c.560delG; p.G187Vfs*60 | No | Yes |
| TP53 p.C135Y, c.404G>A | No | Yes |
| TP53 p.C176Y, c.527G>A | Yes | Yes |
| TP53 p.C238Y | Yes | Yes |
| TP53 p.C242*, c.726_744del | Yes | Yes |
| TP53 p.K132E, c.394A>G | No | Yes |
| TP53 p.M237Ifs*9, c.711_714del | Yes | Yes |
| TP53 p.N310Tfs*35, c.927_928delinsG | No | Yes |
| TP53 p.P223L, c.668C>T | Yes | Yes |
| TP53 p.R248L, c.743G>T | Yes | Yes |
| TP53 p.R273H | Yes | Yes |
| TP53 p.R337C, c.1009C>T | Yes | Yes |
| TP53 p.S241F, c.722C>T | Yes | Yes |

**Table S5.** Coverage of the Oncomine Lung cfDNA assay v1.

| Gene count | 11 |
| --- | --- |
| Gene names | ALK, BRAF, EGFR, ERBB2, KRAS, MAP2K1, MET, NRAS, PIK3CA, ROS1, TP53 |
| Amplicons | 35 |
| Hotspots | 169 |


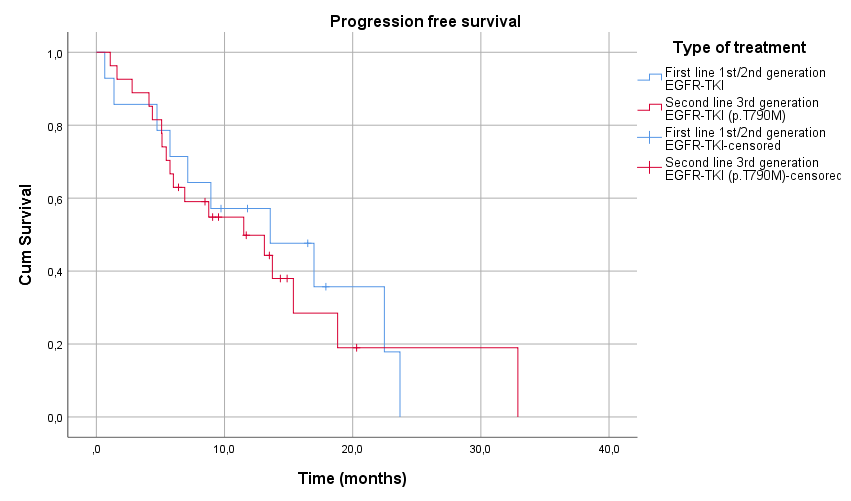


**Figure S1.** PFS in the total *EGFR* cohort according to treatment line. N = 41, *p* = 0.768.


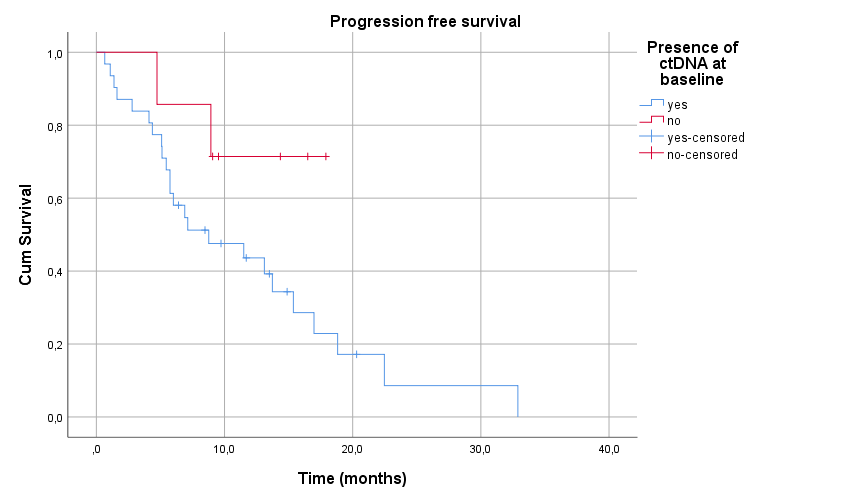


**Figure S2.** PFS in presence or absence of detectable ctDNA (*EGFR* mutations in plasma). N = 38, *p* = 0.108.


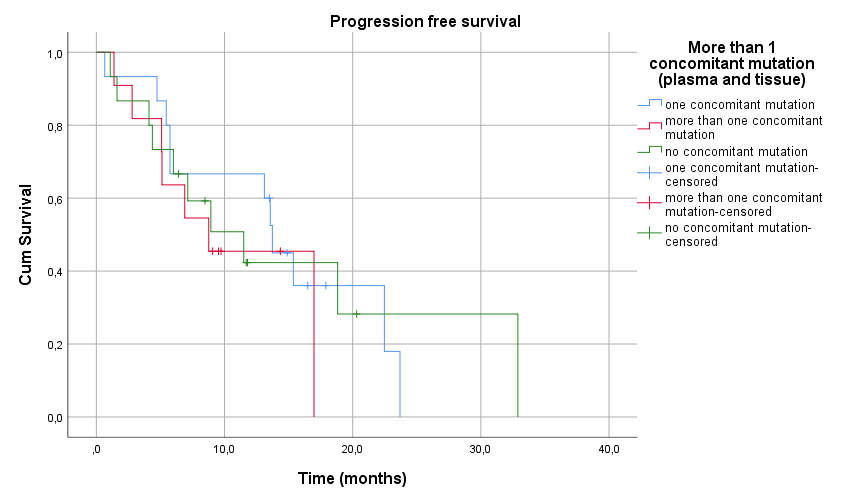


| Concomitant mutations | N= | Events | Median PFS (months) | 95% CI |
| --- | --- | --- | --- | --- |
| 0 | 15 | 10 | 11.5 | 4.6-18.4 |
| 1 | 15 | 11 | 13.7 | 12.7-14.8 |
| >1 | 11 | 7 | 8.8 | 3.3-14.3 |

**Figure S3.** PFS in patients with concomitant mutations (besides *EGFR*). *p* = 0.734.


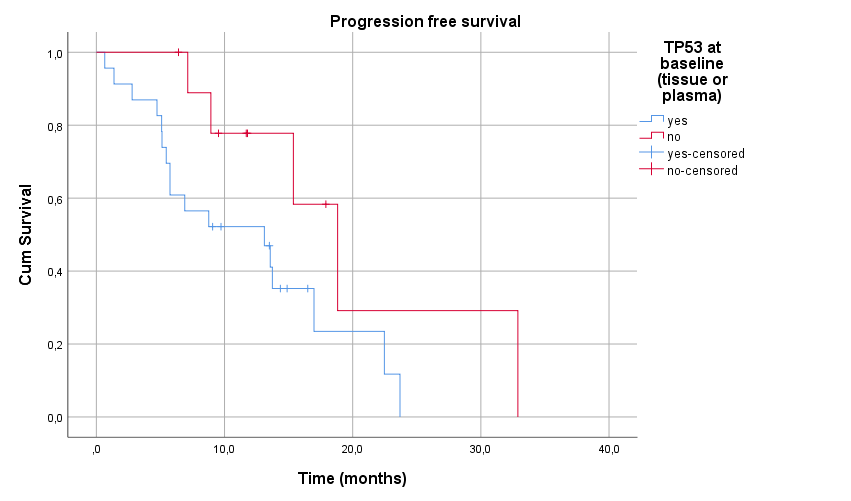


**Figure S4A.** PFS and *TP53* mutational status in the total *EGFR* cohort. N=33, p=0.068.


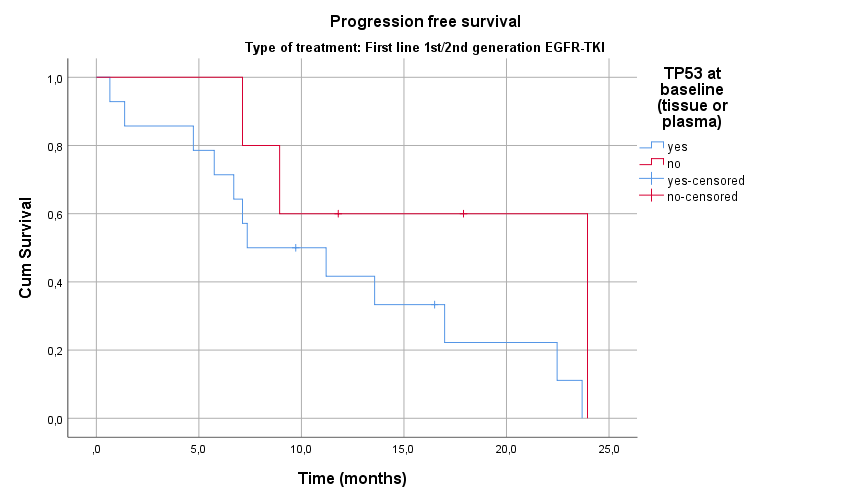


**Figure S4B** PFS and *TP53* mutational status in the first line cohort. N = 19, *p* = 0.116.


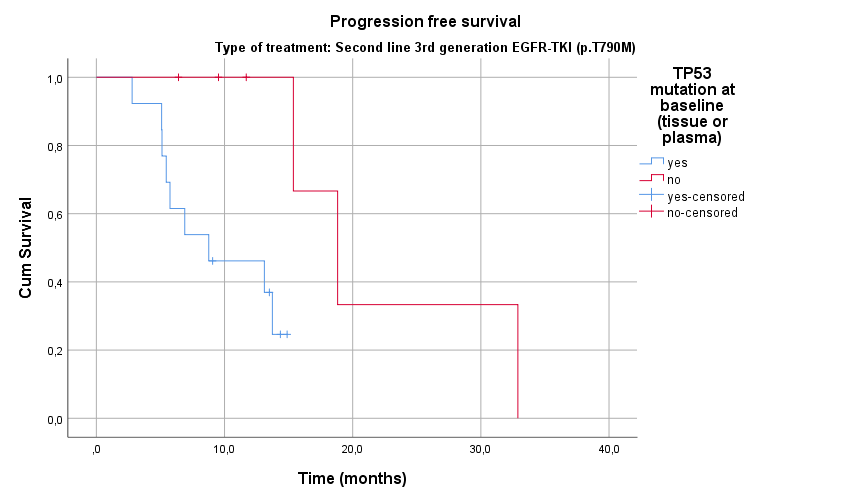


**Figure S4C.** PFS and *TP53* mutational status in the second line cohort. N = 19, *p* = 0.017.


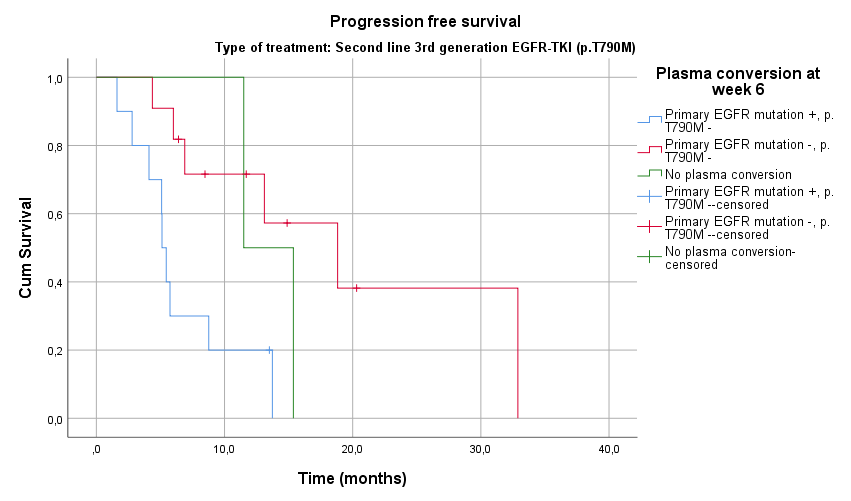


**Figure S5.** PFS and plasma conversion in the second line cohort. N = 23, *p* = 0.012.


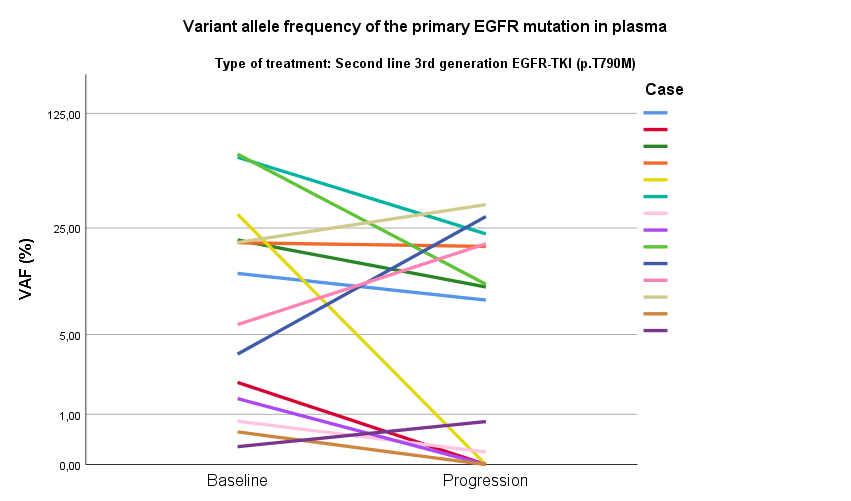


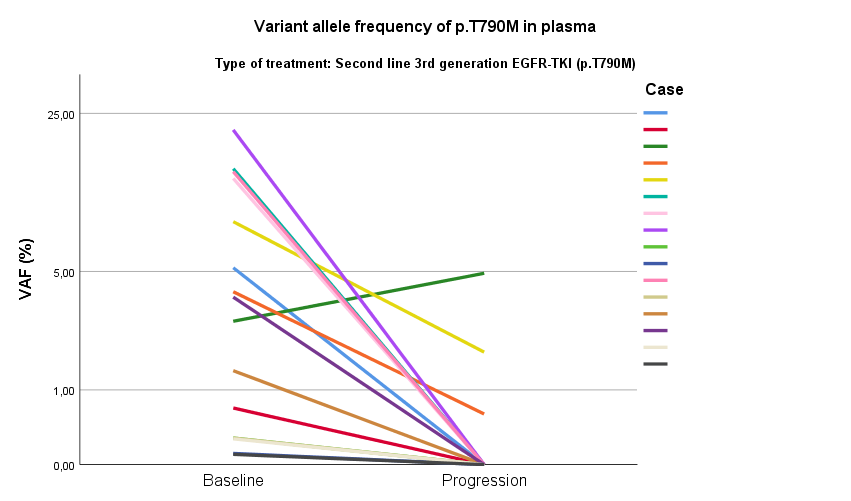


**Figure S6.** Plasma mutation levels in the second line cohort.
